# Essential role for cyclic-AMP responsive element binding protein 1 (CREB) in the survival of acute lymphoblastic leukemia

**DOI:** 10.18632/oncotarget.3911

**Published:** 2015-05-06

**Authors:** Naomi E. van der Sligte, Kim R. Kampen, Arja ter Elst, Frank J.G. Scherpen, Tiny G.J. Meeuwsen-de Boer, Victor Guryev, Frank N. van Leeuwen, Steven M. Kornblau, Eveline S.J.M. de Bont

**Affiliations:** ^1^ Division of Pediatric Oncology/Hematology, Department of Pediatrics, Beatrix Children's Hospital, University Medical Center Groningen, University of Groningen, Groningen, The Netherlands; ^2^ European Research Institute for The Biology of Ageing, University Medical Center Groningen, University of Groningen, Groningen, The Netherlands; ^3^ Laboratory of Pediatric Oncology, Department of Pediatrics, Radboud Institute for Molecular Life Sciences, Radboud University Medical Center, Nijmegen, The Netherlands; ^4^ Department of Leukemia, The University of Texas, MD Anderson Cancer Center, Houston, Texas, United States of America

**Keywords:** acute lymphoblastic leukemia, cyclic-AMP responsive element binding protein, CREB, targeted therapy

## Abstract

Acute lymphoblastic leukemia (ALL) relapse remains a leading cause of cancer related death in children, therefore, new therapeutic options are needed. Recently, we showed that a peptide derived from Cyclic-AMP Responsive Element Binding Protein (CREB) was highly phosphorylated in pediatric leukemias.

In this study, we determined CREB phosphorylation and mRNA levels showing that CREB expression was significantly higher in ALL compared to normal bone marrow (phosphorylation: *P* < 0.0001, mRNA: *P* = 0.004). High CREB and phospho-CREB expression was correlated with a lower median overall survival in a cohort of 140 adult ALL patients. ShRNA mediated knockdown of CREB in ALL cell lines blocked leukemic cell growth by inducing cell cycle arrest and apoptosis. Gene expression array analysis showed downregulation of CREB target genes regulating cell proliferation and glucose metabolism and upregulation of apoptosis inducing genes. Similar to CREB knockdown, the CREB inhibitor KG-501 decreased leukemic cell viability and induced apoptosis in ALL cell lines, as well as primary T-ALL samples, with cases showing high phospho-CREB levels being more sensitive than those with lower phospho-CREB levels.

Together, these *in vitro* findings support an important role for CREB in the survival of ALL cells and identify this transcription factor as a potential target for treatment.

## INTRODUCTION

Acute Lymphoblastic Leukemia (ALL) is the most common pediatric malignancy, comprising almost 25% of all cancers diagnosed among children [1]. Based on immunophenotype, ALL can be classified in two major subtypes; B-cell precursor lineage (BCP-ALL) and T-cell lineage (T-ALL). The clinical outcome of children with ALL remains favorable, with an overall five-year event-free survival rate up to 90% [2]. However, relapse remains common and cure after relapse is difficult to achieve in children [3]. Improvements in outcome obtained over the past few decades are largely attributed to optimization of combination chemotherapy. However, further improvements in outcome appear to be limited by chemotherapy related toxicity [4]. Therefore, treatment approaches using the new generation of available kinase inhibitors targeting leukemia-specific proteins with reduced toxicity may further improve conventional therapeutic strategies.

Recently, we determined kinase activity profiles in primary pediatric ALL, acute myeloid leukemia (AML), and chronic phase chronic myeloid leukemia (CML-CP) samples using kinase activity profiling [5, 6]. These results revealed significantly elevated phosphorylation of a peptide derived from cyclic (c)-AMP responsive element binding protein, phosphorylated on tyrosine 134 and serine 133 (CREB_Y134/S133) in all leukemic samples compared to normal bone marrow (NBM). No significant differences in CREB_Y134/S133 peptide phosphorylation were observed between leukemia subtypes.

The transcription factor CREB acts downstream of major signaling pathways, including cAMP-dependent protein kinase A (PKA), mitogen-activated protein kinases (MAPK) via ribosomal protein S6 kinase (RPS6KA1), and phosphatidylinositol 4, 5-bisphosphate 3-kinase (PI3K) / Akt [7, 8]. After phosphorylation on serine 133, CREB binds to the CREB binding protein (CBP) to regulate the transcription of various target genes involved in cell proliferation, differentiation and survival [8]. In normal hematopoietic cells, previous studies have detected a higher CREB expression in progenitor cells (e.g. CD34^+^ cells) compared to differentiated cells [9]. Numerous studies have investigated the role of CREB in myeloid leukemia [9–12]. Since CREB is frequently expressed in bone marrow cells of patients with both myeloid and lymphoid acute leukemias and below detectable levels in remission samples, it was hypothesized that CREB might regulate essential cell functions in these leukemic cells [9, 10, 13]. Additionally, high CREB expression was found to be associated with an increased risk of AML relapse [10]. CREB downregulation in myeloid leukemia cell lines led to a decrease in cell growth and viability *in vitro* and suppression of leukemia progression followed by a pro-longed overall survival in a murine imatinib resistant CML model [9]. In pediatric AML, it was shown that miR-34b promoter hypermethylation causes CREB overexpression [11]. Hypermethylation of the miR-34b promoter and subsequent higher CREB protein levels were found in 66% of the pediatric AML patients [12]. Despite the multitude of studies into the role of CREB in myeloid leukemias, the relationship between CREB expression levels and outcome and the applicability of CREB as a potential druggable target in ALL has not been examined.

In this study, we have correlated CREB expression in primary ALL patients to outcome. Moreover, we have studied the effects of a loss of CREB function on ALL survival and gene expression profiles using shRNA mediated knockdown of CREB or a CREB inhibitor (KG-501).

## RESULTS

### High CREB expression in pediatric ALL

Previously, using our kinome profiling set, we showed that phosphorylation of a CREB-derived peptide containing residue serine 133, a key residue in the regulation of CREB mediated gene expression, was among the most strongly phosphorylated peptides in the ALL samples [6, 24]. Moreover, the CREB_S133 peptide was more highly phosphorylated in T-ALL compared to BCP-ALL (4995.27 and 3904.16, respectively, *P* = 0.024) [6].

**Figure 1:**
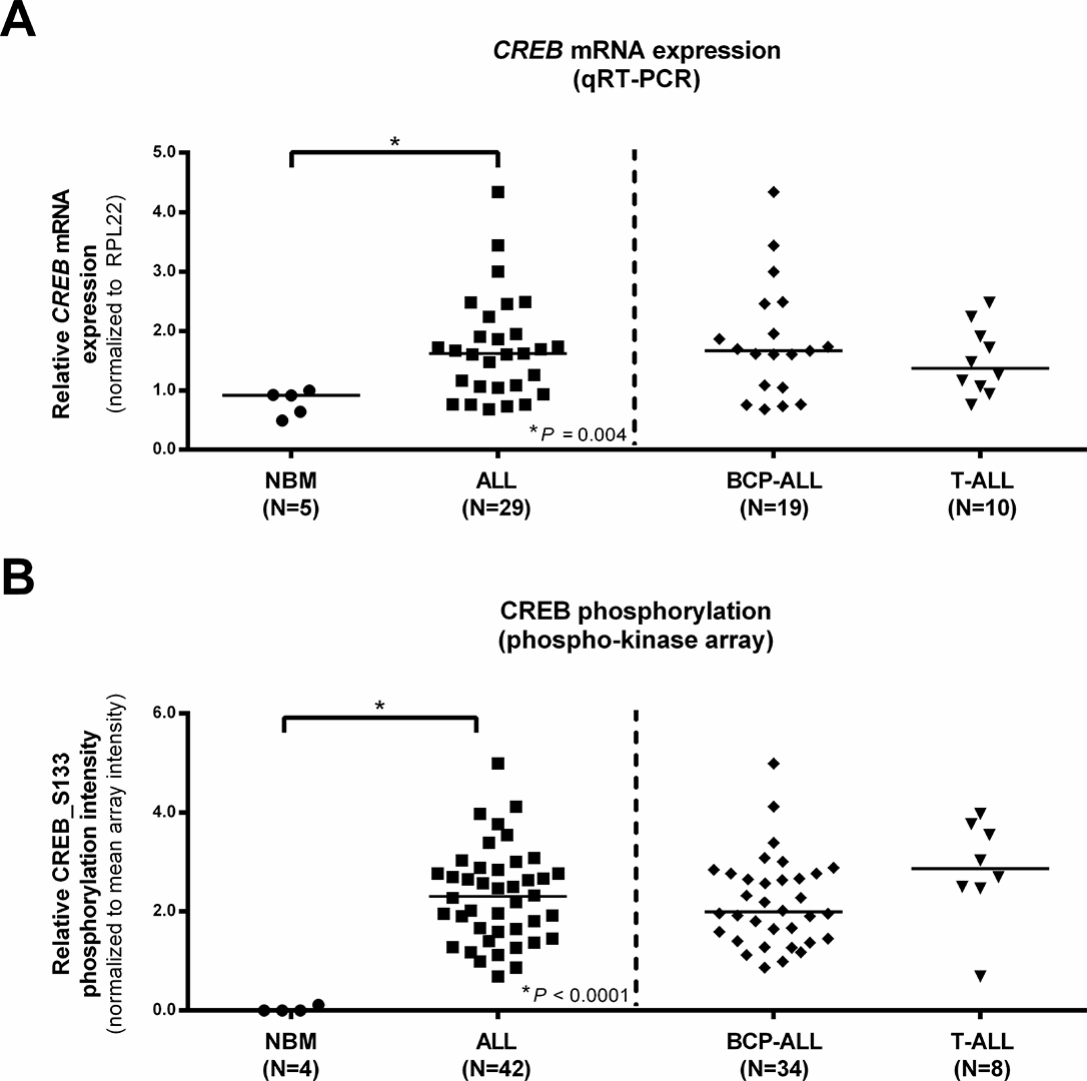
Normalized CREB expression profiles in pediatric ALL and normal bone marrow (NBM) mononuclear cells *CREB* mRNA expression levels for normal bone marrow and ALL patients, ALL patients are displayed as a group and distinguished based on immunophenotype **A.** CREB_S133 phosphorylation determined using phospho-kinase arrays for normal bone marrow and ALL patients. ALL patients are again shown as one group and distinguished based on immunophenotype **B.** Asterisks (*) indicate significant differences (*P* < 0.05) of normal bone marrow vs ALL or BCP-ALL vs T-ALL as determined by the Mann-Whitney *U* test. Horizontal bars indicate median values.

In order to define a role for CREB expression in ALL and to compare the expression levels between T-ALL and BCP-ALL, we first determined *CREB* mRNA levels. Relative *CREB* mRNA levels were consistently higher in ALL compared to normal bone marrow (*P* = 0.004, Figure 1A). *CREB* expression levels did not differ between BCP-ALL and T-ALL (*P* = 0.580, Figure 1A).

Phosphorylation levels of CREB_S133 were analyzed using phospho-kinase arrays. Overall phosphorylation intensities of the 46 protein kinases on the array were higher in the pediatric ALL samples compared to the normal bone marrow samples (Supplementary Figure 1 and Supplementary Table 4). Consistent with earlier reports, a prominent difference in phosphorylation intensities between normal bone marrow and leukemia samples was observed for CREB_S133 phosphorylation (Figure 1B) [13]. Normalized CREB_S133 phosphorylation intensities showed an 83-fold increase in primary pediatric ALL compared to normal bone marrow (mean normalized phosphorylation intensities of 2.32 and 0.028, respectively, *P* < 0.0001, Figure 1B). Phosphorylation intensities did not differ between T-ALL and BCP-ALL (2.84 and 2.19, respectively, *P* = 0.069, Figure 1B). As expected, we observed no correlation between *CREB* mRNA expression levels and CREB_S133 phosphorylation intensities (r_s_ = 0.142, *P* = 0.529).

### High CREB expression and phosphorylation is associated with reduced overall survival in adult primary ALL blasts

**Figure 2:**
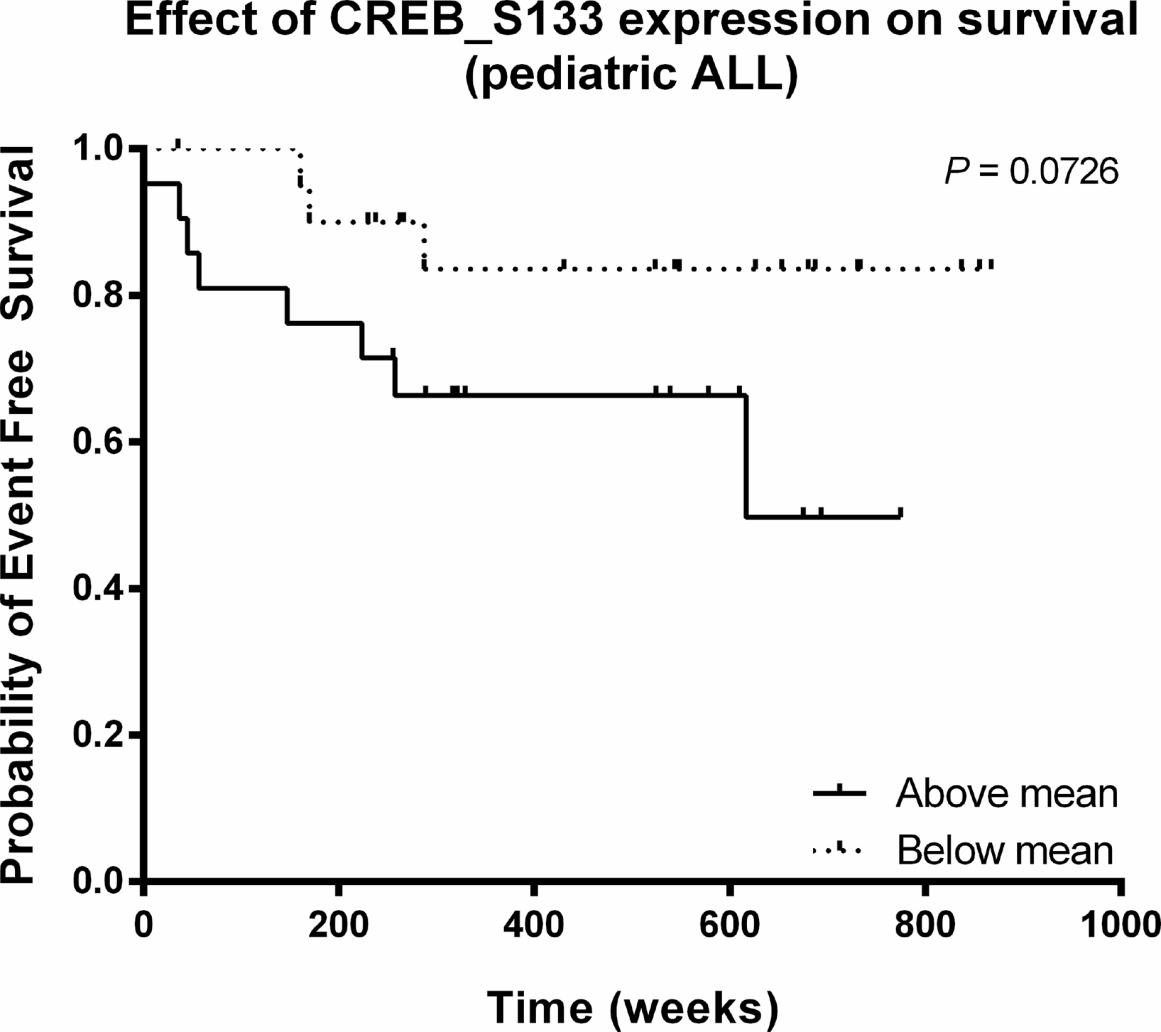
Relationship between CREB phosphorylation and event-free survival in pediatric ALL Kaplan-Meier plot of event-free survival time as function of CREB phosphorylation. The estimated event-free survival time appeared to be lower for patients with high CREB phosphorylation intensities (*P* = 0.073).

As a high CREB expression was found to be associated with poor outcome in AML, we investigated the relationship between estimated event-free survival rates and CREB phosphorylation for 42 pediatric ALL patients. Patients were divided into two groups based on CREB phosphorylation levels (above and below mean CREB phosphorylation intensities). In spite of the small number of patients, we observed that patients with high CREB phosphorylation levels trended towards a lower event-free survival relative to patients with low phosphorylation levels (*P* = 0.073, Figure 2).

**Figure 3:**
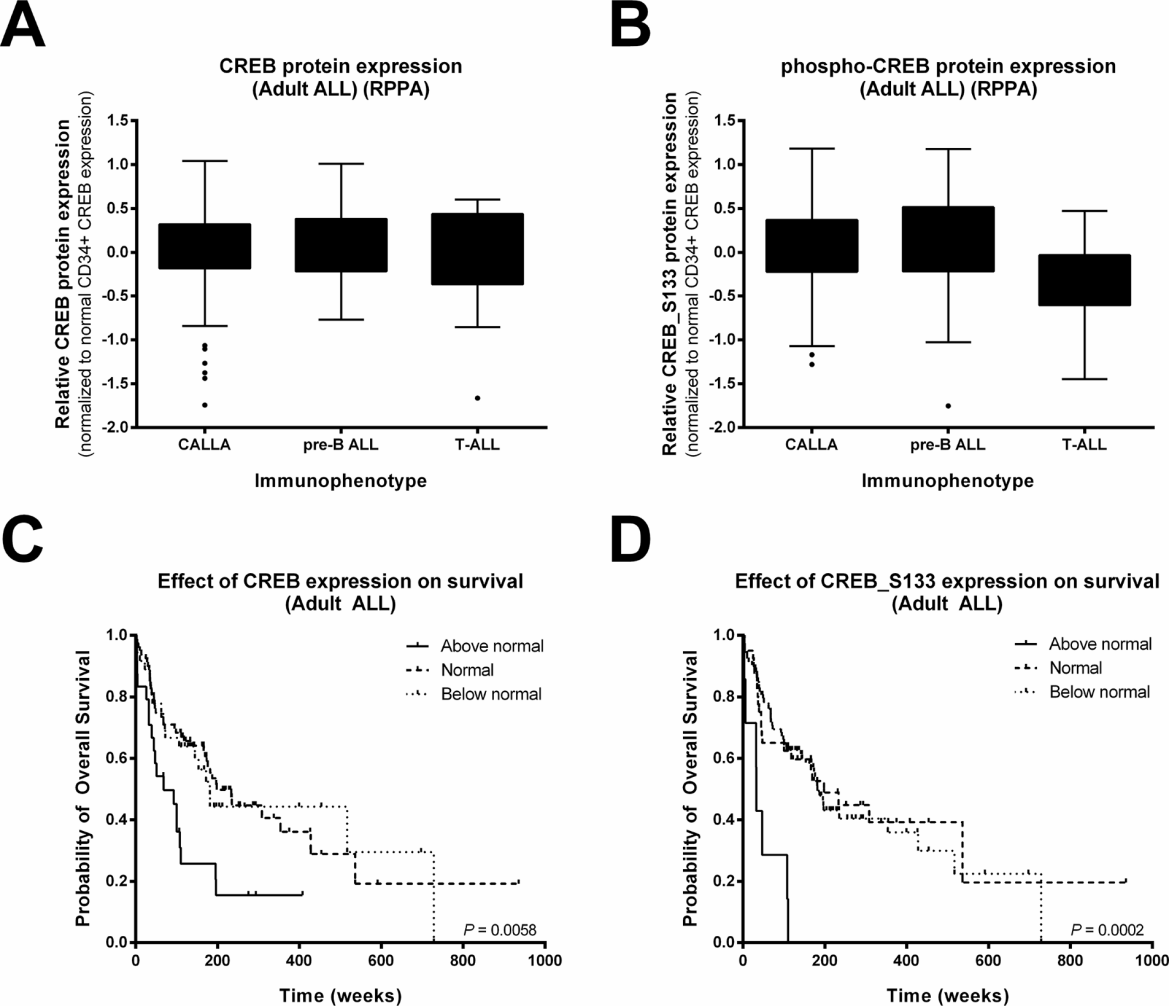
High CREB expression and phosphorylation is associated with reduced overall survival in adult ALL Box-plots of CREB **A.** and phospho-CREB **B.** protein levels of 140 Ph-negative adult ALL patients distinguished based on immunophenotype. Expression levels were normalized using normal CD34^+^ cells as a comparison because of their known high CREB expression. To determine the overall survival, patients were divided into three groups based on CREB **C.** and phospho-CREB **D.** expression levels. The proportion of surviving patients was lower for patients with high CREB or phospho-CREB expression levels compared to patients with an expression at normal CD34^+^ levels (*P* = 0.002 and *P* = 0.001, respectively) or below normal CD34^+^ levels (*P* = 0.015 and *P* < 0.001, respectively)

As we observed a trend in our small pediatric cohort, we interrogated a previously described reverse phase protein array (RPPA) data set of 140 newly diagnosed mostly young adult ALL patients (Figure 3, Supplementary Table 5A and 5B) [15]. In this dataset, normal bone marrow CD34^+^ cells were used as comparison since these cells are known to have high CREB expression levels [9]. CREB expression and CREB_S133 phosphorylation was found to be above that of normal CD34^+^ cells in 17.1% and 5.0% of the ALL patient samples, respectively, while 25.7% (CREB) and 66.4% (phospho-CREB_S133) of the primary ALL samples showed expression below that of normal bone marrow CD34^+^ cells (Figure 3A and 3B, Supplementary Table 5A). To investigate the prognostic value of CREB and phospho-CREB expression levels, we examined the relationship between overall survival rates and CREB expression and phosphorylation. Patients were divided into three groups based on expression levels (below normal, normal, and above normal protein expression levels compared to the 90 interpercentile of normal bone marrow CD34^+^ protein expression levels). Although CREB_S133 protein phosphorylation was high in only 7 patients, both high CREB and phospho-CREB_S133 levels were found to be associated with a poor outcome compared to normal or below normal expression levels (Figure 3C and 3D). Patients with high CREB expression or phospho-CREB_S133 levels showed a lower median overall survival (68 and 33 weeks, respectively) compared to patients with normal levels (CREB and phospho-CREB_S133 both 198 weeks, *P* = 0.002 and *P* = 0.001, respectively) or below normal levels (181 and 182 weeks, *P* = 0.015 and *P* < 0.001, respectively, Figure 3C and 3D). We conclude from these results that high expression or activity of CREB is associated with an unfavorable prognosis in ALL.

### shRNA-mediated downregulation of CREB reduces cell growth and cell viability

**Figure 4:**
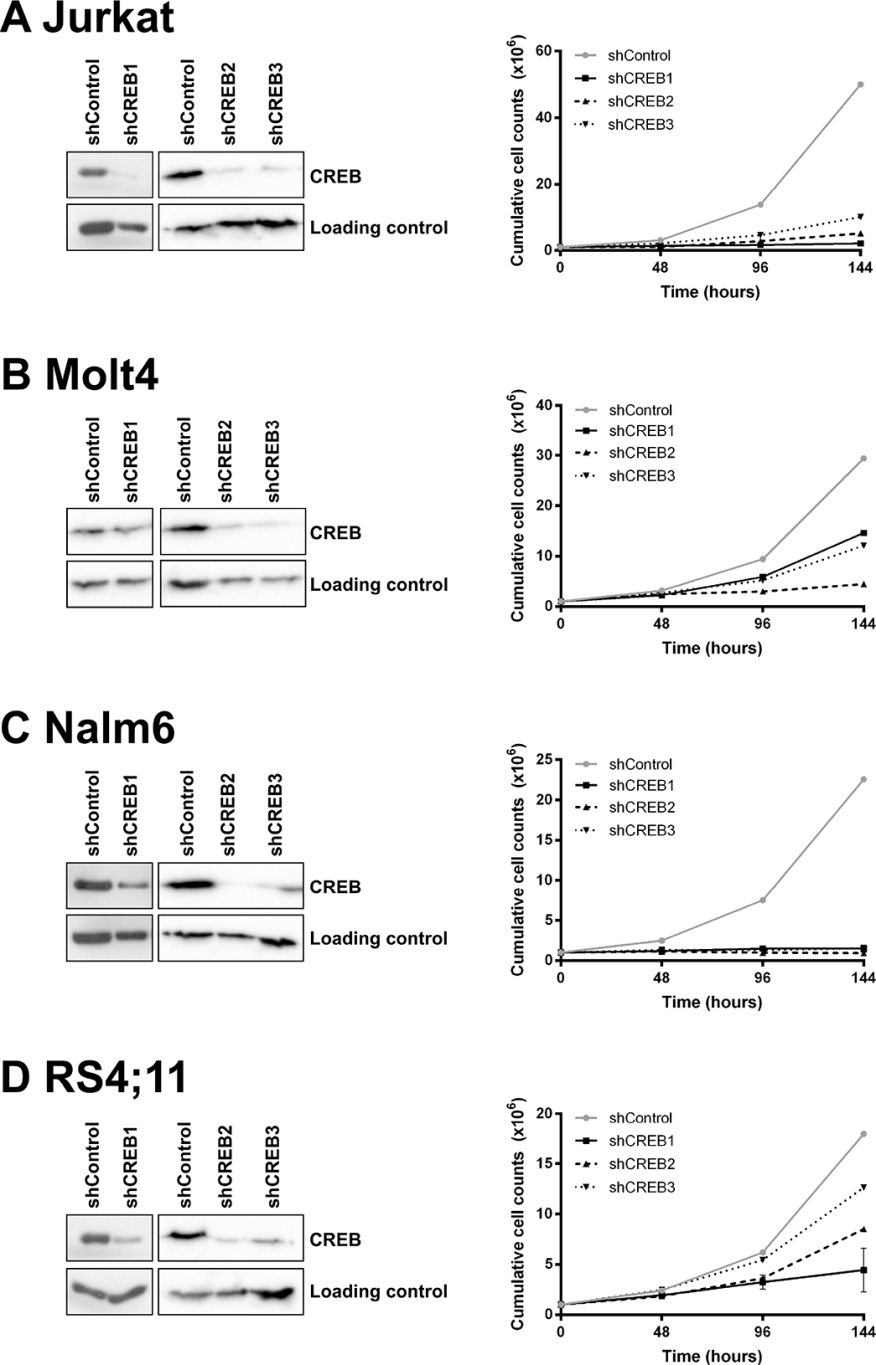
CREB is essential for leukemic cell growth and viability CREB is successfully downregulated in 4 ALL cell lines using three different CREB shRNAs. Downregulation was confirmed by Western blot analysis as shown in de left panels. Growth curve analysis shows that CREB downregulation results in a decrease in cell growth (right panels). Growth was measured in duplicate by absolute cell counts every 48 hours using a Coulter Counter. The results are shown as mean ± standard deviation.

Since CREB expression and activity appears to be higher in ALL cells compared to normal bone marrow, CREB may represent an important signaling protein both in pediatric and adult ALL. Hence, we investigated the role of CREB on leukemic cell survival performing shRNA mediated knockdown of CREB in four ALL cell lines using three different shRNAs. CREB protein expression levels were reduced in all cell lines, albeit with variable efficiencies. Despite multiple transductions, shCREB1, while highly effective in the other cell lines, produced only a minimal reduction in expression (85% of shControl cells) in the Molt4 cells. In all other cases, a reduction in protein expression levels ≤ 55% of the shControl transduced cells was observed (Figure 4, left panels). Moreover, we observed that CREB knockdown effectively impaired leukemic cell growth in these four cell lines ranging from 30% in RS4;11 up to 96% in Jurkat and Nalm6 (Figure 4, right panels).

**Figure 5:**
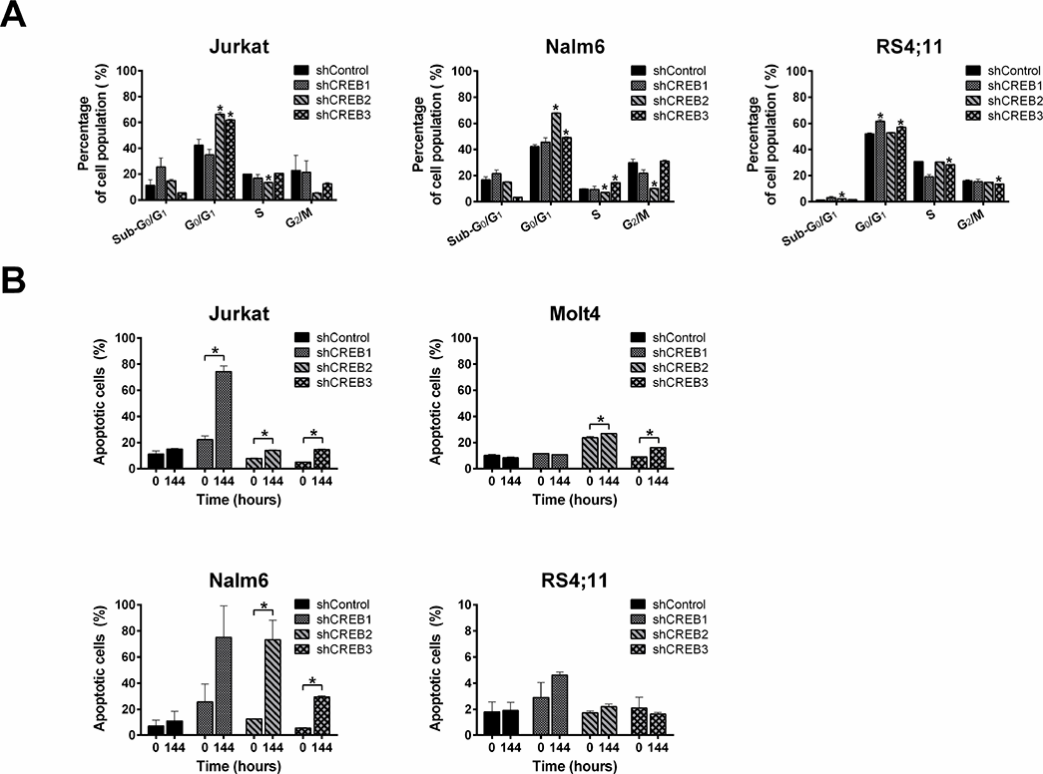
CREB downregulation induces cell cycle arrest and apoptosis Cell cycle analysis shows a reduction in the percentage of cells in S and G_2_/M phases with a concomitant increase of cells in sub-G_0_ and G_0_/G_1_ phases in comparison with shControl **A.** Annexin-V staining shows an increase in apoptotic cell numbers for CREB downregulated cells as compared to control transduced cells **B.** The results are shown as mean ± standard deviation. Asteriks (*) indicate significant differences (*P* < 0.05) by Student *t*-test.

To explain the dramatic effect of CREB knockdown on cell growth, we studied the consequences of CREB knockdown on cell cycle progression and apoptosis. Cell cycle analysis for Jurkat, Nalm6, and RS4;11 revealed that CREB knockdown decreased the percentage of cells in S and G_2_/M phases with a concomitant increase of cells in the apoptotic sub-G_0_/G_1_ and / or G_0_/G_1_ phases, consistent with a cell cycle arrest (Figure 5A). No cell cycle analysis was performed on Molt4 transduced cells since this is an aneuploid cell line. Complementary to the increase of cells in the apoptotic sub-G_0_/G_1_ phase, Annexin-V staining showed an increase in apoptotic cell numbers for the shCREB transduced cells at the 144 hour time point as compared to the 0 hour time point, which was not observed in cells transduced with a scrambled shRNA control (Figure 5B).

### Gene expression profiling of CREB knockdown cells

Gene expression profiling was performed to examine genetic consequences that resulted from shRNA mediate knockdown of CREB in the four ALL cell lines Jurkat, Nalm6, Molt4, and RS4;11 using shCREB2 and shCREB3. Since CREB was shown to act either as a transcriptional activator (when phosphorylated) or as a repressor, we were interested in genes that were either up- or downregulated [25]. We identified 1, 022 genes that were significantly up- or downregulated in both shCREB2 and shCREB3 versus shControl transduced cells (*P* < 0.05, Supplementary Table 6). To identify more specific CREB target genes within the list of differentially expressed genes we correlated differences in gene expression to the amount of CREB knockdown. Of the 535 upregulated genes, 510 genes were inversely correlated with CREB protein expression, and 463 genes were correlated to the 487 downregulated genes (Pearson's correlation, *P* < 0.05, Supplementary Table 6). The list of downregulated genes included putative CREB target genes regulating cell proliferation and cell cycle e.g. *CIAO2, CSNK1A1, MCM10, PAFAH1B1, PSMD5, STAT6, TFDP1, UBE2V1*, and *UBE2V2*. Of the known CREB target genes we observed upregulation of *BAD, BNIP2,* and *DEDD2*, genes implicated in the induction of apoptosis.

**Figure 6:**
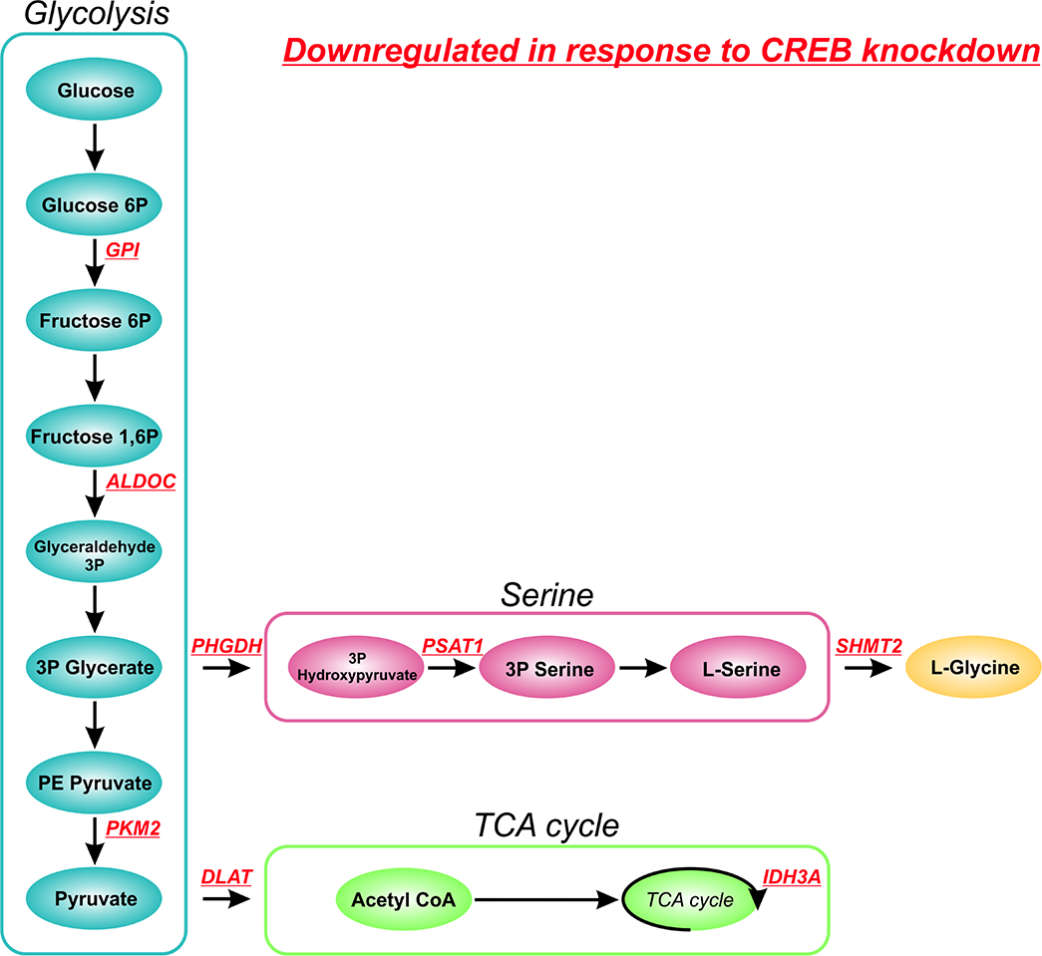
Effect of CREB knockdown on glucose metabolism Schematic overview of metabolic enzymes involved in glucose metabolism affected by shRNA mediated CREB knockdown. Downregulated genes, as determined by gene expression profiling, and their role in glucose metabolism are shown in red.

Interestingly, KEGG pathway analysis identified the metabolic pathway involved in glucose metabolism as a key effector of CREB knockdown. Multiple genes involved in glycolysis e.g. *GPI, ALDOC,* and *PKM2*, tricarboxylic acid cycle (TCA cycle) e.g. *DLAT* and *IDH3A* and serine and glycine biosynthesis e.g. *PHGDH, PSAT1*, and *SHMT2* were observed in the list of downregulated genes (Figure 6). Since it has been previously shown that glucose metabolism plays a central role in sustaining cancer growth and oncogenic transformation [26], the downregulation of essential genes involved in this pathway likely contributes to the observed phenotypic effects observed in response to CREB knockdown.

### Decreased ALL cell survival in response to the CREB inhibitor KG-501

**Figure 7:**
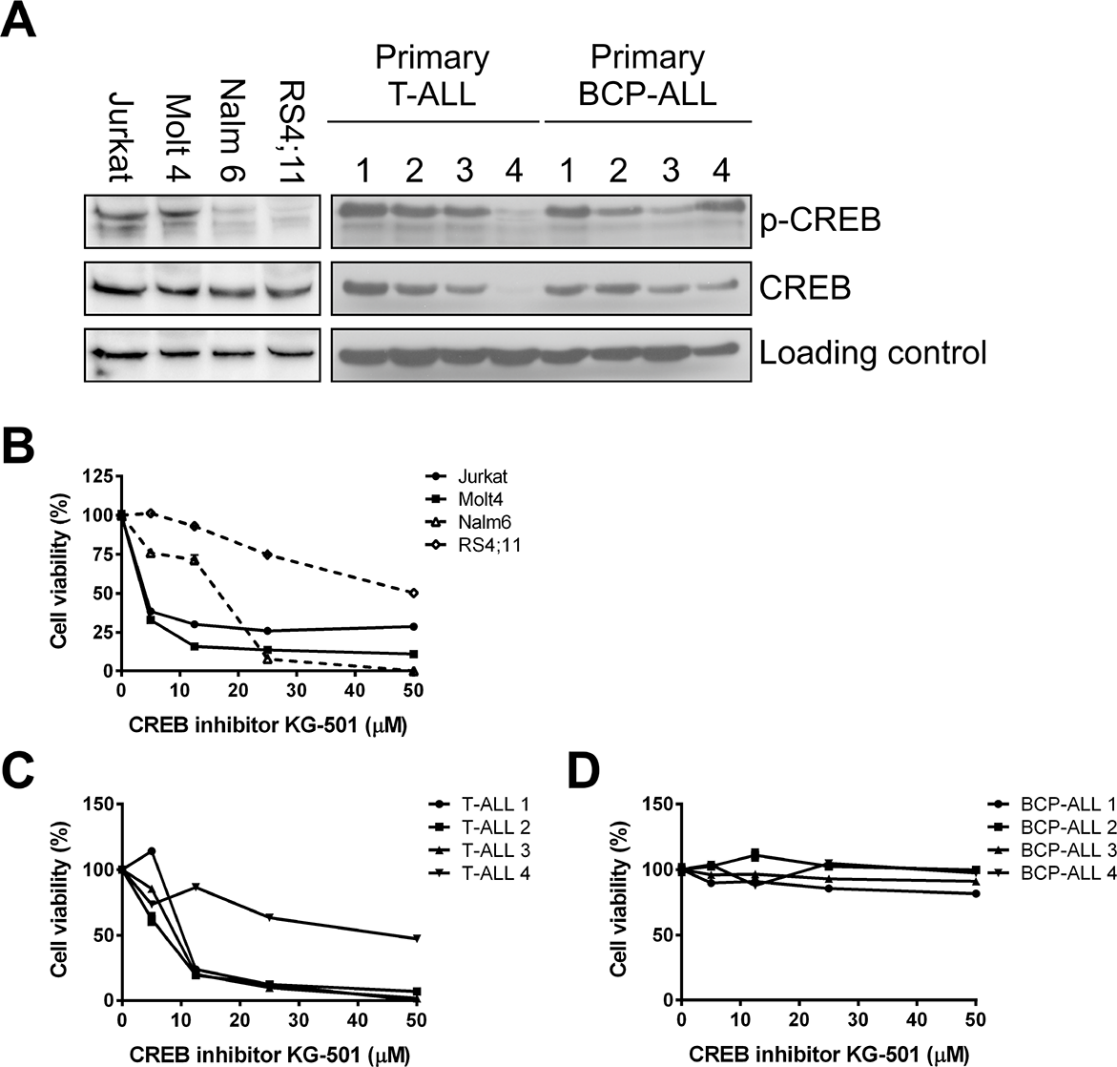
Inhibition of CREB induces cell death in a dose-dependent manner CREB and phospho-CREB expression was determined by Western blot analysis for BCP-ALL and T-ALL cell lines and primary samples **A.** Effects of the CREB inhibitor KG-501 were determined after 24 or 48 hours of incubation for primary samples and cell lines, respectively. CREB inhibition results in a dose-dependent decrease in cell viability as determined by WST-1 conversion. Cell viability percentages were plotted against concentration of KG-501 (μM) using four ALL cell lines **B.**, four primary BCP-ALL samples **C.**, and four primary T-ALL samples **D.**

The effects of pharmacological inhibition on leukemic cell survival was evaluated using the CREB inhibitor KG-501 in 4 ALL cell lines and 8 primary pediatric ALL patient samples. KG-501 restricts the function of CREB by inhibiting the CREB/CBP interaction, which requires phosphorylation of CREB at serine 133. CREB_S133 expression levels in the 4 ALL cell lines and 8 primary ALL patient samples are shown (Figure 7A). Exposure to KG-501 induced a dose-dependent decrease in cell viability in all cell lines tested, but the LC-50 values for the T-ALL cell lines Jurkat (4.1 μM) and Molt4 (3.7 μM) were lower than those for the BCP-ALL cell lines Nalm6 (16.7 μM) and RS4;11 (>50 μM). This was largely concordant with the effects seen after CREB knockdown (Figure 7B). Interestingly, RS4;11 cells, which were least affected by CREB knockdown and KG-501 treatment, showed the lowest phospho-CREB expression levels (Figure 7A). The observed decrease in cell viability was inversely correlated with the percentage of apoptotic cell numbers (r_s_ = −0.67, *P* = 0.017, Supplementary Figure 2).

Next, the effects of the KG-501 inhibitor were evaluated in 8 primary pediatric ALL samples. Whereas none of the BCP-ALL samples responded to the drug despite high CREB phosphorylation, all 4 primary T-ALL samples showed sensitivity to the drug in a dose-dependent manner (Figure 7C and 7D, LC-50 values for T-ALL 1, 2, 3, and 4 were 10.32 μM, 7.16 μM, 9.07 μM, and 45.90 μM, respectively). The differences in sensitivity were reflected by CREB and phospho-CREB levels, with primary leukemic T-ALL cells showing high CREB expression or activity (T-ALL samples no. 1, 2, and 3) being more sensitive than those with low CREB and phospho-CREB levels (T-ALL sample no. 4) (Figure 7A).

## DISCUSSION

Although the survival of children with ALL is approaching 90%, ALL relapse remains a leading cause of cancer-related death in children [1, 2]. To further improve clinical outcome, new therapeutic strategies are necessary. To date, the effects of CREB inhibition and its role in leukemogenesis have been primarily studied in acute myeloid leukemia. Here, we report a prominent role for CREB in ALL. Our results show that, relative to normal bone marrow, CREB is overexpressed both at the mRNA and protein level, consistent with previous results of Pigazzi et al, showing high expression of CREB in 94% pediatric ALL cases [13]. Additionally, CREB has frequently undergone an activating phosphorylation on serine 133. No correlation between *CREB* mRNA expression levels and CREB phosphorylation could be observed in our study. Since protein phosphorylation is the net result of intracellular kinase activity and basal protein expression levels (influenced by mRNA translation and protein degradation) and the generally poor correlation between protein and mRNA concentrations, this may not be surprising [27]. Our results demonstrate also that high expression and / or phosphorylation of CREB are associated with a lower overall survival in an adult ALL patient cohort. Furthermore, CREB appears to be essential for leukemic cell growth, since both CREB shRNA mediated knockdown and pharmacological inhibition leads to growth inhibition and cell death.

Studying CREB knockdown using shRNAs in ALL cell lines resulted in decreased cell numbers that appeared to be net result of changes in cell cycle distribution and the induction of apoptosis. We performed gene expression profiling to elucidate the phenotypic effects observed in response to shRNA mediated CREB knockdown. First of all we observed effects of CREB knockdown on CREB target genes implicated in cell proliferation, cell cycle, and apoptosis, consistent with the observed phenotypic effects. Interestingly however, within the list of downregulated genes, enzymes involved in glycolysis (*GPI, ALDOC*, and *PKM2*), TCA cycle (*DLAT* and *IDH3A*), and serine and glycine biosynthesis (*PHGDH, PSAT1*, and *SHMT2*) were among the most prominent. Although *DLAT* and *IDH3A* were previously described as putative CREB target genes, all genes were listed as genes with predicted functional CREs [28]. The role of CREB in glucose metabolism has been extensively studied, especially in the context of diabetes mellitus, showing a central role for transducers of regulated CREB activity (TORCs, especially TORC2) in CREB-dependent gene transcription in response to glucose [29, 30]. In cancer cells, an increase in glycolysis, known as the Warburg effect, is frequently observed, which allows the generation of essential precursors for the synthesis of proteins, nucleic acids, and lipids from glycolytic intermediates [31, 32]. The observed downregulation of these genes in response to shRNA mediated CREB knockdown contributes to the strong phenotypic effects observed in our cell lines.

In addition to shRNA mediated knockdown, we also explored the effects of the CREB inhibitor KG-501. We previously showed that cells from normal bone marrow are insensitive to the KG-501 inhibitor [14]. In accordance with the CREB phosphorylation levels and the shRNA results, the cell lines Jurkat, Molt4, Nalm6, and 3 / 4 primary T-ALL samples were sensitive to CREB inhibition. Unexpectedly, while shRNA mediated CREB knockdown and CREB inhibition showed similar phenotypic effects in T-ALL cell lines and primary T-ALL samples, we observed different phenotypic effects between BCP-ALL cell lines and primary samples using pharmacological CREB inhibition. Although shRNA mediated CREB knockdown, as well as KG-501 showed corresponding results in BCP-ALL cell lines, KG-501 had no effect on the survival of primary BCP-ALL samples, despite high CREB phosphorylation levels. Although human cancer derived cell lines are the most widely used models to test therapeutic strategies, the limitations of cell line models are well known [33, 34]. Additionally, this discrepancy may be explained by CREB target gene regulation. Transcription of CREB target genes is partly dependent on CREB phosphorylation at serine 133 and the subsequent CREB/CBP interaction. CREB target gene transcription may also be induced in a phosphorylation independent manner by a family of CREB coactivators, of which TORC complexes are the most potent (Supplementary Figure 3) [35]. The small molecule antagonist KG-501 restricts the function of CREB by inhibiting the CREB/CBP interaction in a dose-dependent way (Supplementary Figure 3) [36]. Therefore, sensitivity to the CREB/CBP interaction inhibitor KG-501 appears to be only observed when the transcription of CREB target genes is predominantly dependent on the CREB/CBP interaction. We hypothesize that cells unresponsive to KG-501 treatment are able to maintain mediated transcriptional regulation by recruiting coactivators that act independent of this CREB/CBP interaction.

Moreover, it is also possible that primary T-ALL cells are more dependent on CREB relative to BCP-ALL cells. The cAMP response element (CRE) has been found in the promoters of many T cell-specific genes [37, 38]. Furthermore, it has been shown that phosphorylation of the proto-oncogene VAV1, downstream of the T cell and pre-T cell receptor, activates CREB and that *in vivo* VAV1-deficiencies cause T cell developmental defects that resemble CREB-deficiency [39]. Moreover, this CREB dependency might be Notch 1 dependent. Aberrant activation of the Notch 1 signaling pathway is seen in > 60% of the T-ALL cases [40]. Notch 1 is able to activate the serine/threonine kinase Akt, which acts upstream of CREB, contributing to leukemogenesis [41]. Although no direct relationship between Notch 1 and CREB has been reported in leukemia to date, Notch regulates the phosphorylation of CREB involved in long-term memory formation in *Drosophila melanogaster* [42]. Finally, we cannot rule out that the difference between primary T-ALL and BCP-ALL samples reflects KG-501 off-target effects. Since the presence of the KIX domain is required for more transcriptional activities, other inhibitory effects of KG-501 on factors that associate with the KIX domains, such as NF-kB, have been observed [36]. In addition, a recent study describes that KG-501 also interferes with the interaction between c-Myb and the KIX domain of the coactivator p300 *in vitro* and inhibits Myb activity *in vivo* [43].

In conclusion, we have demonstrated that high CREB expression and activity is associated with reduced overall survival in ALL. Moreover, our *in vitro* findings suggest an important role for CREB in the survival of acute lymphoblastic leukemia cells. These results underscore the need for future investigations aimed at validating the role of CREB as a potential therapeutic target for acute lymphoblastic leukemia.

## MATERIALS AND METHODS

### Patients

Primary blood and bone marrow samples from ALL patients or patients without hematologic disorders were collected after getting written informed consent in accordance with the regulations and protocols of the Medical Ethical Committee of the University Medical Center Groningen (UMCG) and the Institutional Review Board of the University of Texas M.D. Anderson Cancer Center (MDACC). Samples from UMCG were processed as previously described [14]. Patients’ characteristics, as well as an overview of the selected ALL patients is shown (Supplementary Tables 1 and 2, respectively). For RPPA analysis we used protein derived from the leukemia-enriched fraction of 140 mostly young adult ALL patients (79 Philadelphia-negative common-ALL (CALLA), 42 Philadelphia-negative preB-ALL, and 19 T-ALL) [15]. As cytogenetic abnormalities are differentially distributed between pediatric and adult patients, we excluded the *BCR-ABL1* positive leukemias and the B-Cell lymphoma population from the adult cohort. Patients’ characteristics of adult ALL patients are summarized (Supplementary Table 3).

### Cell lines

The T-ALL cell lines Jurkat and Molt4 and the BCP-ALL cell lines Nalm6 and RS4;11 were purchased from the American Type Culture Collection (Manassa, VA, USA) and cultured in RPMI-1640 or αmem medium (Cambrex Bio Science, East Rutherford, NJ, USA) supplemented with 10% fetal calf serum (FCS, Bodinco, Alkmaar, The Netherlands) and 1% penicillin/streptomycin (Gibco, Carlsbad, CA, USA).

### RNA isolation and quantitative real-time PCR (qRT-PCR)

Total RNA was isolated (RNeasy mini kit, Qiagen, Hilden, Germany) and subsequent complementary DNA was synthesized from 1 μg RNA (First Strand cDNA Synthesis kit, Thermo Scientific, Waltham, MA, USA) according to manufactures protocol. *CREB* mRNA expression was detected in triplicate using SYBR Green qRT-PCR (Applied Biosystems, Darmstadt, Germany) and normalized to RPL22 as a reference gene [16]. Relative mRNA expression levels of the gene of interest were calculated using the 2^−ΔΔCT^ method relatively to the normal bone marrow sample with the highest *CREB* mRNA expression level [17].

### Antibody microarrays

Acute lymphoid leukemia patient samples were subjected to a human phospho-kinase microarray (Catalog Number ARY003, R&D Systems, Minneapolis, MN, USA) following manufacturers protocol. Eight of the 42 arrays (4 T-ALL and 4 BCP-ALL) were published before [6]. The experimental procedures and data analysis were performed as described earlier [6].

### Reverse phase protein array (RPPA)

The methodology, validation, and statistical analysis of the RPPA technique are fully described in previous publications and are briefly summarized in the Supplementary Information [18–23]. Expression was compared to the expression of normal CD34^+^ cells as a well-defined control with a known high CREB expression. Cases were divided into three cohorts, below, above or within the 90% confidence interval of normal CD34^+^ cells, respectively.

### shRNA mediated knockdown

Lentiviral vector (pLKO.1) containing shRNA sequences targeting CREB were obtained from Open Biosystems (Waltham, MA, USA) and genetically modified into a pLKO1-mCherry vector. Sequences are available upon request. Lentiviral particles were generated by co-transfection of pLKO1-mCherry-shCREB or scrambled vector with packaging plasmid psPAX2 and the envelope plasmid pMD2.G into 293T cells using FuGENE HD transfection reagent (Roche, Woerden, The Netherlands). Leukemic cells were incubated with lentiviral supernatants for one or two consecutive days after which stably transduced cells were expanded.

### Western blot analysis

Cells from primary blood or bone marrow samples from newly diagnosed ALL patients, ALL cell lines, and transduced cells were lysed and denaturized in laemmli sample buffer (Bio-Rad laboratories, Veenendaal, The Netherlands). Western blots were performed as previously described [14]. CREB and phospho-CREB antibodies were obtained from Cell Signaling (Danvers, MA, USA). Loading control was visualized using β-actin (Santa Cruz Biotechnology, Dallas, TX, USA) or Ponceau S (Sigma-Aldrich, Buchs, Switzerland). Signals were digitally quantified with subtraction of background (LI-COR Biosciences, Lincoln, NE, USA) and normalized using the loading control.

### Flow cytometric analysis

Apoptosis analysis was conducted by Annexin V-FITC/PI or Annexin V-FITC labeling solution (Annexin-V-FLUOS Staining Kit, Roche). A propidium iodide/Rnase A DNA labeling was performed for cell cycle analysis according to standard techniques as described in the Supplementary Information. All flow cytometry experiments were performed on a LSR-II flow cytometer (BD FACS DIVA software, BD bioscience, Breda, The Netherlands) and analyzed using FlowJo software (Tree Star Inc., Ashland, OR, USA).

### Gene expression

Total RNA was isolated from CREB or scrambled transduced cells (RNeasy mini kit). The quality control, RNA labeling, hybridization, and data extraction was profiled on Illumina HumanHT-12 v4.0 Expression BeadChips, all performed at ServiceXS B.V. (Leiden, The Netherlands). Scans (Illumina iScan), image analysis and extraction of raw data (Illumina GenomeStudio v2011.1) was done, after which we performed quantile normalization and log2-transformation. Gene expression data have been deposited in NCBI's Gene Expression Omnibus (GEO) database and are accessible through GEO Series accession number GSE68413.

### Cell viability assay

*In vitro* drug sensitivity after KG-501 (Sigma-Aldrich) exposure was determined with a WST-1 based cell viability assay (Roche) as described previously, and calculated as percentage of untreated controls [14]. LC50 values represent drug doses inhibiting cell viability within 24 or 48 hours.

### Statistical analysis

Estimated event-free and overall survival analyses were performed using the Kaplan-Meier method in SPSS (Version 20, IBM, Armonk, NY, United States). Estimates at specific time points are provided with 95% confident intervals (CIs). Data was censored at time of an event or at the last follow-up visit. The logrank test was applied to compare Kaplan-Meier plots between patient groups. Experimental data is presented as mean values from in general three experiments ± standard deviation (SD), unless described differently. Statistical analyses were perfromed with The non-parametric Mann-Whitney *U* test and the parametic paired and unpaired two-tailed *t*-test (SPSS). *P*-values < 0.05 were considered as statistically significant.

## SUPPLEMENTARY MATERIALS AND METHODS FIGURES AND TABLES




