# Supplementary material for: Essential role for cyclic-AMP responsive element binding protein 1 (CREB) in the survival of acute lymphoblastic leukemia
Source: Oncotarget. 2015 May 6;6(17):14970–81. doi: 10.18632/oncotarget.3911 (PMC4558129; doi:10.18632/oncotarget.3911)
Supplement: Supplementary file 1 [file oncotarget-06-14970-s001.pdf]

SUPPLEMENTARY MATERIALS AND METHODS

Reverse phase protein array (RPPA)

Patient samples were printed onto slides in 5 serial dilutions along with controls for normalization and expression. Slides were probed with strictly validated 1:1000 diluted primary antibodies against total CREB (Catalog Number 1296–1, LOTC08024, Epitomics, Burlingame, CA, USA) or CREB phosphorylated on serine 133 (Catalog Number 1113–1, LOTYE09240–1, Epitomics). A secondary antibody (1:40 dilution) was used to amplify the signal, and a stable dye was precipitated. The stained slides were analyzed using Microvigene® software (Version 3.0, Vigene Tech, Carlisle, MA, USA) to produce quantified data.

Statistical analysis: Supercurve algorithms were used to generate a single value from the 5 serial dilutions [20]. The loading control and topographical normalization procedures accounted for variations in protein concentration and background staining [21, 22]. Analysis using unbiased clustering, perturbation bootstrap clustering a principle component analysis were performed as fully described in previous publications [19, 23].

Cell cycle analysis

Cells were pelleted and fixated in cold absolute methanol and stored at –20°C for at least 20 minutes then washed and stained in phosphate-buffered saline (PBS) containing 1% bovine serum albumin (BSA), 1 mg/ml RNAse A and 50 µg/ml PI for 40 min at 37°C. Analysis of DNA content was performed on a flow cytometer (LSR-II, BD FACS DIVA software, BD bioscience) and data was analyzed using FlowJo software (Tree Star Inc.).

SUPPLEMENTARY FIGURES AND TABLES

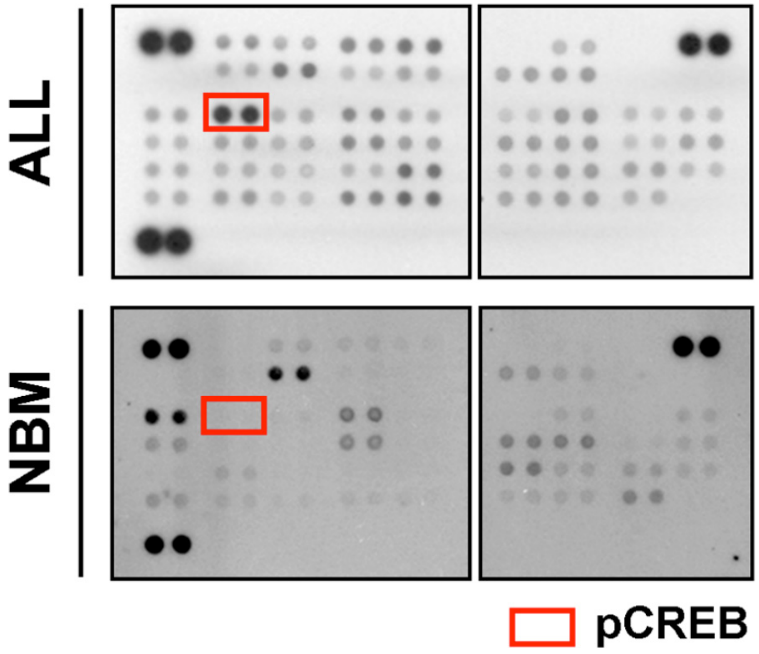

Supplementary Figure 1: A representative image of a human proteome profiler array for an ALL sample and a normal bone marrow sample, the CREB signal is highlighted in red.

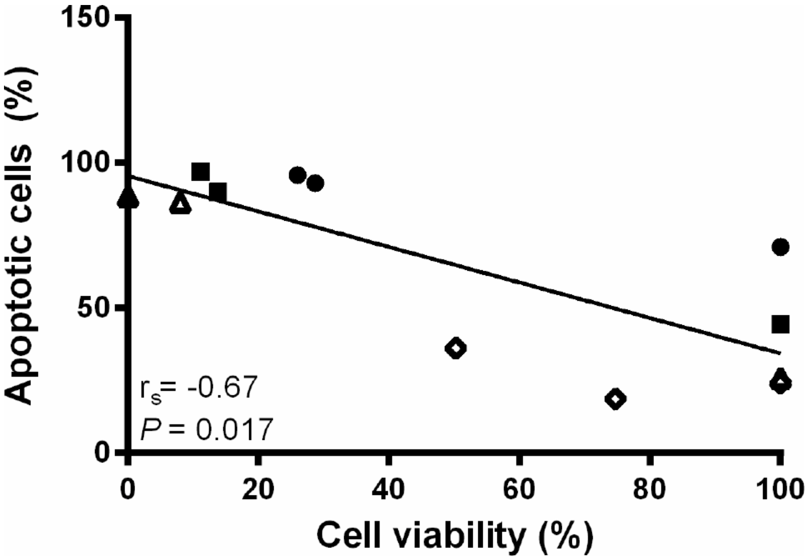

Supplementary Figure 2: Effects of the CREB inhibitor KG-501 on apoptosis and cell viability in ALL cell lines measured by AnnexinV/PI staining and WST-conversion after 48 hours. The increase in apoptosis was inversely correlated to the decrease in cell viability. Diagonal line represents the best fit. The correlation coefficient was determined by the non-parametric Spearman's rank (r_s) test.

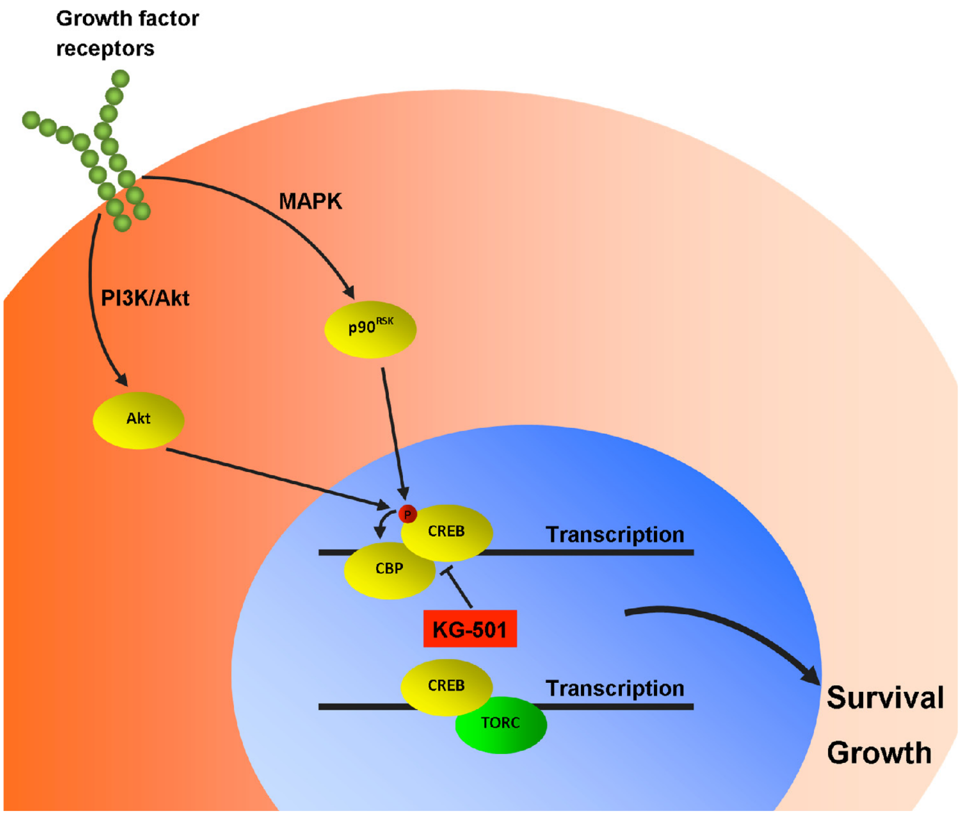

Supplementary Figure 3: Schematic overview of CREB target gene transcription (adapted from KM Sakamoto et al. [8]).

Supplementary Table 1: Patients' characteristics of pediatric patients

Demographics of Clinical Characteristics	*No. of patients (N = 55)*	*%*
**Age, years**		
Median (range)	9.1 (1.8–16.2)	
**Immunophenotype**		
BCP-ALL	44	80.0%
T-ALL	11	20.0%
**Cytogenetics**		
BCP-ALL		
Normal karyotype	13	29.5%
t(1;19)	3	6.8%
t(12;21)	1	2.3%
t(4;11)	1	2.3%
t(9;22)	1	2.3%
Hyperdiploid	6	13.6%
Down syndrome	3	6.8
Other	12	27.3%
Unknown	4	9.1%
T-ALL		
Normal karyotype	6	54.5%
t(10;11)	1	9.1%
Other	4	36.4%

Abbreviations: BCP-ALL; B-cell progenitor ALL

Supplementary Table 2: Overview of selected ALL patients. A plus "+" indicates that material of the patient is used for the afore mentioned experiments. Material of patients not used for specific experiments is indicated with a minus "–".

	De Novo / Relapse	Cytogenetics	qRT-PCR	Kinase profile	Phospho-kinase array	Western blot	*In vitro* experiments
BCP-ALL 1	De Novo	Hyperdiploid	+	–	–	+	+
BCP-ALL 2	De Novo	Normal	+	–	–	+	+
BCP-ALL 3	De Novo	t(4;11)	+	–	–	+	+
BCP-ALL 4	De Novo	Other	+	–	–	+	+
BCP-ALL 5	De Novo	Normal	+	+	+	–	–
BCP-ALL 6	De Novo	Normal	–	+	–	–	–
BCP-ALL 7	De Novo	t(1;19)	+	+	+	–	–
BCP-ALL 8	De Novo	Hyperdiploid	+	+	+	–	–
BCP-ALL 9	De Novo	Other	+	–	+	–	–
BCP-ALL 10	De Novo	Other	+	–	+	–	–

(*Continued*)

	De Novo / Relapse	Cytogenetics	qRT-PCR	Kinase profile	Phospho-kinase array	Western blot	*In vitro* experiments
BCP-ALL 11	De Novo	Normal	+	+	+	–	–
BCP-ALL 12	De Novo	Normal	+	+	+	–	–
BCP-ALL 13	De Novo	Unknown	+	+	+	–	–
BCP-ALL 14	De Novo	Normal	+	+	+	–	–
BCP-ALL 15	De Novo	Other	+	–	+	–	–
BCP-ALL 16	De Novo	Other	+	–	+	–	–
BCP-ALL 17	De Novo	Unknown	+	–	+	–	–
BCP-ALL 18	De Novo	Other	+	–	+	–	–
BCP-ALL 19	De Novo	Normal	+	–	+	–	–
BCP-ALL 20	De Novo	Hyperdiploid	–	+	+	–	–
BCP-ALL 21	De Novo	Normal	+	–	+	–	–
BCP-ALL 22	De Novo	Normal	–	+	–	–	–
BCP-ALL 23	De Novo	Unknown	–	+	–	–	–
BCP-ALL 24	De Novo	Normal	–	+	–	–	–
BCP-ALL 25	De Novo	Normal	–	+	–	–	–
BCP-ALL 26	De Novo	t(1;19)	–	+	–	–	–
BCP-ALL 27	De Novo	Other	–	–	+	–	–
BCP-ALL 28	De Novo	Other	–	–	+	–	–
BCP-ALL 29	De Novo	Down	–	–	+	–	–
BCP-ALL 30	De Novo	Hyperdiploid	–	–	+	–	–
BCP-ALL 31	De Novo	Other	–	–	+	–	–
BCP-ALL 32	De Novo	Unknown	–	–	+	–	–
BCP-ALL 33	De Novo	t(1;19)	–	–	+	–	–
BCP-ALL 34	De Novo	Other	–	–	+	–	–
BCP-ALL 35	De Novo	Normal	–	–	+	–	–
BCP-ALL 36	De Novo	Other	–	–	+	–	–
BCP-ALL 37	De Novo	t(9;22)	–	–	+	–	–
BCP-ALL 38	De Novo	Down	–	–	+	–	–
BCP-ALL 39	De Novo	Other	–	–	+	–	–
BCP-ALL 40	De Novo	t(12;21)	–	–	+	–	–
BCP-ALL 41	De Novo	Down	–	–	+	–	–
BCP-ALL 42	De Novo	Normal	–	–	+	–	–
BCP-ALL 43	De Novo	Hyperdiploid	–	–	+	–	–
BCP-ALL 4	De Novo	Hyperdiploid	–	–	+	–	–
T-ALL 1	De Novo	Other	+	+	+	+	+
T-ALL 2	De Novo	Other	+	–	–	+	+
T-ALL 3	De Novo	Other	+	–	–	+	+

(*Continued*)

	De Novo / Relapse	Cytogenetics	qRT-PCR	Kinase profile	Phospho-kinase array	Western blot	*In vitro* experiments
T-ALL 4	De Novo	Other	+	–	–	+	+
T-ALL 5	De Novo	Normal	+	+	+	–	–
T-ALL 6	De Novo	Normal	+	+	+	–	–
T-ALL 7	De Novo	Normal	–	+	+	–	–
T-ALL 8	De Novo	t(10;11)	+	–	+	–	–
T-ALL 9	De Novo	Normal	+	–	+	–	–
T-ALL 10	De Novo	Normal	+	+	+	–	–
T-ALL 11	De Novo	Normal	+	+	+	–	–

Supplementary Table 3: Patients' characteristics of adult patients

Demographics of Clinical Characteristics	*No. of patients (N =)*	*%*
**Age, years**		
Median (range)	35.5 (1.1–83)	
**Immunophenotype**		
CALLA	79	56.4%
preB-ALL	42	30.0%
T-ALL	19	15.6%
**Cytogenetics**		
BCP-ALL		
Diploid	35	28.9%
Hyperdiploid	16	13.2%
Hypodiploid	2	1.7%
t(1;19)	1	0.8%
t(4;11)	10	8.3%
Other	36	29.8%
Unknown	21	17.4%
T-ALL		
T-ALL	7	36.8%
ETP	4	21.1%
Mature	2	10.5%
Unknown	6	31.6%

Abbreviations: CALLA; Common-ALL, BCP-ALL; B-cell progenitor ALL, ETP; early T cell progenitor

Supplementary Table 4: Mean normalized protein phosphorylation of the 46 kinases spotted on the human phospho-kinase microarray for ALL and NBM samples

Peptides	ALL (*N* = 24)		NBM (*N* = 4)	
	Mean	*SD*	*Mean*	*SD*
Akt_S473	0, 72	0, 17	0, 06	0, 12
Akt_T308	1, 38	0, 35	1, 72	0, 47
AMPKa1	0, 58	0, 26	0, 27	0, 22
AMPKa2	1, 34	0, 38	2, 89	0, 54
b-Catenin	0, 50	0, 24	0, 09	0, 19
Chk-2	1, 38	0, 81	0, 78	0, 33
c-Jun	1, 18	0, 27	1, 05	0, 20
CREB	2, 55	0, 97	0, 03	0, 06
eNOS	0, 97	0, 22	1, 38	0, 85
ERK 1/2	0, 63	0, 46	1, 26	0, 85
Fak	0, 49	0, 17	0, 31	0, 10
Fgr	0, 49	0, 30	0, 01	0, 02
Fyn	0, 78	0, 29	0, 26	0, 18
GSK-3ab	1, 50	0, 59	0, 46	0, 19
Hck	0, 71	0, 35	0, 84	0, 37
HSP27	1, 04	0, 40	0, 35	0, 11
JNKpan	1, 05	0, 38	0, 67	0, 45
Lck	0, 48	0, 20	0, 03	0, 06
Lyn	0, 95	0, 21	0, 16	0, 21
MEK	0, 95	0, 21	0, 30	0, 26
MSK 1/2	1, 35	0, 28	3, 24	2, 59
mTOR	0, 91	0, 34	4, 10	2, 32
p27_T157	0, 60	0, 15	0, 17	0, 12
p27_T198	0, 71	0, 55	0, 01	0, 03
p38a	1, 02	0, 54	0, 03	0, 07
p53_S15	1, 19	0, 27	1, 12	0, 75
p53_S392	1, 08	0, 55	0, 13	0, 15
p53_S46	1, 37	0, 20	0, 95	0, 32
p70 S6 kinase_T229	0, 70	0, 32	2, 69	0, 59
p70 S6 kinase_T389	0, 44	0, 26	0, 00	0, 00
p70 S6 kinase_T421/S424	1, 23	0, 24	2, 50	0, 54
Paxillin	0, 85	0, 16	0, 63	0, 16
PLCg-1	0, 90	0, 27	0, 71	0, 10

(*Continued*)

Peptides	ALL (N = 24)		NBM (N = 4)	
	Mean	*SD*	*Mean*	*SD*
Pyk2	0, 74	0, 19	0, 60	0, 17
RSK 1/2	1, 30	0, 28	0, 91	0, 08
RSK 1/2/3	1, 28	0, 28	1, 88	0, 50
Src	0, 82	0, 22	4, 60	3, 70
STAT1	1, 06	0, 22	0, 60	0, 09
STAT2	1, 30	0, 35	2, 98	0, 84
STAT3	0, 59	0, 13	0, 03	0, 07
STAT4	1, 02	0, 53	0, 72	0, 11
STAT5a	0, 77	0, 17	0, 53	0, 83
STAT5a/b	1, 53	0, 30	0, 29	0, 41
STAT5b	1, 29	0, 77	0, 62	0, 96
STAT6	1, 36	0, 40	1, 13	1, 10
Yes	0, 93	0, 31	0, 69	0, 20

Supplementary Table 5: Description of CREB and phoshpo-CREB expression in various cell subtypes using RPPA. Above normal, normal and above normal protein expression levels are compared to the 90 interpercentile of NBM $CD34^{+}$ protein levels.

A CREB Expression

							Percentages		
	Mean	SD	Median	Min	Max	Obs Number	> Normal expression	Normal expression	< Normal expression
Calla	0.004	0.533	0.081	−1.743	1.042	79	16.46%	59.49%	24.05%
Pre-B	0.066	0.418	0.097	−0.770	1.011	42	19.05%	52.38%	28.57%
T-ALL	0.004	0.589	0.159	−1.663	0.601	19	15.79%	57.89%	26.32%

B CREB_S133 Expression

							Percentages		
	Mean	SD	Median	Min	Max	Obs Number	> Normal expression	Normal expression	< Normal expression
Calla	0.065	0.530	0.152	−1.279	1.182	79	5.06%	30.38%	64.56%
Pre-B	0.138	0.548	0.226	−1.752	1.179	42	7.14%	35.71%	57.14%
T-ALL	−0.370	0.468	−0.368	−1.447	0.473	19	0.00%	5.26%	94.74%

Supplementary Table 6: List of up- and downregulated genes in both shCREB 2 and CREB3 versus shControl transduced cells (Student's *t*-test, *P* < 0.05). Genes significantly (inversely) correlated with CREB knockdown are cursive and bold highlighted (Pearson's correlation, *P* < 0.05).
